# Novel Oligo-Ester-Ether-Diol Prepared by Waste Poly(ethylene terephthalate) Glycolysis and Its Use in Preparing Thermally Stable and Flame Retardant Polyurethane Foam

**DOI:** 10.3390/polym11020236

**Published:** 2019-02-01

**Authors:** Cuong N. Hoang, Chi T. Pham, Thu M. Dang, DongQuy Hoang, Pyoung-Chan Lee, Soo-Jung Kang, Jinhwan Kim

**Affiliations:** 1 Department of Polymer Chemistry, University of Science, Vietnam National University, 227 Nguyen Van Cu, Ho Chi Minh City 70000, Vietnam; hncuong@hcmus.edu.vn; 2 Department of Polymer and Composite Materials, University of Science, Vietnam National University, 227 Nguyen Van Cu, Ho Chi Minh City 70000, Vietnam; phamchi0404@gmail.com (C.T.P.); dtmthu.hcmus@gmail.com (T.M.D.); 3 Department of Polymer Science and Engineering, Sungkyunkwan University, Gyeonggi 440-746, Korea; soojung@skku.edu; 4 Lightweight Materials R&D Center, Korea Automotive Technology Institute, Chonan, Chungnam 31214, Korea; pclee@katech.re.kr

**Keywords:** oligo-ester-ether-diol, recycled poly(ethylene terephthalate) bottle, flame-retardant polyurethane foam, thermal properties, cone calorimeter, flame-retardant mechanism

## Abstract

Rigid polyurethane foam (PUF) was successfully prepared from a novel oligo-ester-ether-diol obtained from the glycolysis of waste poly(ethylene terephthalate) (PET) bottles via reaction with diethylene glycol (DEG) in the presence of ZnSO_4_·7H_2_O. The LC-MS analysis of the oligodiol enabled us to identify 67 chemical homologous structures that were composed of zero to four terephthalate (T) ester units and two to twelve monoethylene glycol (M) ether units. The flame retardant, morphological, compression, and thermal properties of rigid PUFs with and without triphenyl phosphate (TPP) were determined. The T_g_ values showed that TPP played a role of not only being a flame retardant, but also a plasticizer. PUF with a rather low TPP loading had an excellent flame retardancy and high thermal stability. A loading of 10 wt % TPP not only achieved a UL-94 V-0 rating, but also obtained an LOI value of 21%. Meanwhile, the PUF without a flame retardant did not achieve a UL-94 HB rating; the sample completely burned to the holder clamp and yielded a low LOI value (17%). The fire properties measured with the cone calorimeter were also discussed, and the results further proved that the flame retardancy of the PUF with the addition of TPP was improved significantly. The polymeric material meets the demands of density and compression strength for commercial PUF, as well as the needs of environmental development. The current study may help overcome the drawback of intrinsic high flammability and enlarge the fire safety applications of materials with a high percentage of recycled PET.

## 1. Introduction

Polyurethane is an important specialty polymer. When polyfunctional isocyanates and intermediates react with polyols or diols in proper ratios, a polymer that can produce rigid or flexible foams, elastomers, coatings, adhesives, and sealants is formed [1,2,3]. Although only a few isocyanates have been commercialized, there are several polyols, namely, polyethers, polyesters, etc. This results in a wide range of polyurethane materials. Varying properties of polyurethane can be customized by choosing the right polyol components.

Poly(ethylene terephthalate) (PET) is a major engineering thermoplastic that shows high tensile and mechanical strength and is dimensionally stable. PET has many important applications, especially in the production of single-serve soft drink and water bottles, thin films, and textiles. These bottles and thin films become waste material after usage; consequently, PET recycling by physical or chemical methods is essential.

Chemical recycling is one of the most acceptable methods among the technologically feasible recycling techniques that follow the principles of sustainable development, because it can form monomers/oligomers from which a new polymer is prepared. One of the most investigated approaches to chemically recycling PET is glycolysis with diols, such as ethylene glycol, diethylene glycol (DEG), triethylene glycol, propylene glycol, dipropylene glycol [4], 1,4-butanediol [5], and poly(ethylene glycol) [6].

A catalyst is a key factor that affects the rate of PET glycolysis by DEG, and various catalysts have been used. Common and effective catalysts include acetate salts of zinc [7], manganese [8,9], tin [9], lead [10], and titanium isopropoxide [11]. Another important factor is the initial molar ratio of DEG/PET, which ranges from 1/1 [7] to 2.2/1 [8].

The glycolysis of PET with conventional heating normally requires a high temperature and long reaction time, e.g., 2 h at 240 °C [7] or 8 h at 190–200 °C [10]. For that reason, microwave irradiation can be applied to shorten the reaction time. For instance, PET glycolysis was performed in a sealed microwave reactor, in which the pressure and temperature were controlled [12], and in a modified domestic microwave oven at 600 W [13] and 450 W [14]. Furthermore, the reactions of various catalysts, namely, manganese acetate [12], zinc acetate [13,14], sodium bicarbonate and sodium carbonate [13], with molar ratios of DEG/PET from 2/1 [14] to 10/1 [12] and reaction times from 6 min [13] to 30 min [14] were reported.

The product of PET glycolysis is a low molecular weight oligodiol, and this diol has been used as a starting material for polyurethane preparation. P.K. Roy et al. [14] conducted chemical recycling of PET waste with diethylene glycol (DEG) using DEG/PET molar ratios of 2/1, 4/1 and 6/1 in the presence of Zn(OAc)_2_·2H_2_O (0.5 wt % of PET) under microwave irradiation at 450 W. The glycolyzed product from the 4/1 ratio was further reacted with adipic acid and sebacic acid to obtain aromatic oligoester diols for polyurethane-polyisocyanurate foam preparation by reaction with MDI. This multistep, complex procedure used only a small quantity of PET waste for the final PU formulation, and therefore is not efficient for PET recycling.

The reaction of recycled PET waste with trimethylolpropane or pentaerythritol was performed by Atta et al. [15] The oligomers were reacted with 2,4-toluene diisocyanate to produce PUF. However, the costs of polyol reactants are quite high; therefore, such recycling of PET is not cost effective.

Additionally, PET textile wastes were depolymerized by a large excess of ethylene glycol (EG/PET molar ratio of 9.3/1) and zinc acetate dihydrate as the catalyst to form bis(2-hydroxyethyl) terephthalate (BHET) [16]. The BHET was repeatedly recrystallized from water, and the large excess of used EG became waste material. The subsequent reaction of BHET diol with methylene diphenyl diisocyanate (MDI) was performed to create polyurethane foam (PUF). The density of the PUF decreased from 510 kg/m^3^ to 150 kg/m^3^ as the water content increased from 3.0% to 6.5%. The density of the PUF was quite high compared to that of commercial PUF. The authors used dimethyl methylphosphonate (DMMP) as a flame retardant in the PUF, and the LOI of DMMP-PUFs approached 27.69% with increasing concentrations of DMMP.

Another PUF was prepared from a polyol obtained from depolymerized PET and liquefied wood polyesters [17]. Commercial PET was glycolyzed with PEG400 (a PEG400/PET molar ratio of 2/1) using a dibutyltin oxide catalyst at 215 °C for 150 min. The reaction was further treated with adipic acid to form copolyester diols. Mixtures of polyester diols and liquefied wood polyesters were reacted with MDI (NCO/OH = 1.7/1) to form PUFs, and the T_g_ values of the PUFs were reported as 58.9 °C to 66.1 °C. The DSC curves from this research showed that these T_g_ values overlapped with a large endothermic peak for moisture evaporation at 95 °C.

To use an oligodiol obtained from the glycolysis of PET, especially in coatings, the color and physical state of the oligodiol is very important. These characteristics can be controlled by using various catalysts and different initial diol/PET molar ratios. 

The zinc acetate catalyst has been used quite often and is effective in PET glycolysis. However, the oligodiol product usually has a dark color. In addition, the oligodiol obtained from the PET-DEG reaction is partly soluble in water; therefore, product purification and catalyst removal or recovery are almost impossible. In our research paper [18], we found that ZnSO_4_·7H_2_O was significantly better than the commonly used zinc acetate for PET glycolysis. The reactions were conducted under microwave irradiation, for effective energy usage, with a low initial DEG/PET ratio, and the liquid product exhibited a bright color. The oligodiol product was partially lost by treatment with a cool 20% NaCl solution, and therefore, the isolation yield was rather low (approximately 36–38%).

In this research project, we used a simple purification step to remove only the solid catalyst. This oligodiol was characterized and used directly for rigid PUF preparation, with and without TPP as a flame retardant.

Rigid PUF has a high surface-to-volume ratio, and a major drawback is its high combustibility; therefore, it demands the addition of a flame retardant. Triphenyl phosphate (TPP) has found some use in rigid foam formulations. A blend of triethyl phosphate and triphenyl phosphate, Lanxess Levagard^®^ TPP, is used in rigid polyurethane and isocyanurate foams, particularly in Europe [19]. However, until now, no publication investigating the flame retardancy of rigid PUFs derived from PET recycled with DEG has been reported. The presence of aromatic rings in the oligodiol obtained from glycolyzed PET could enhance the thermal stability and flammability of PUF, and consequently, the quantity of TPP could be reduced. Cone calorimeter, compression test, differential scanning calorimetry, and thermogravimetric analyses of rigid PUF with and without TPP are also discussed.

## 2. Materials and Methods 

### 2.1. Materials

Caps and labels were removed from waste, colorless PET bottles before washing with water, cutting into 5 × 5 mm^2^ flakes, and drying in an oven at 80 °C for 1 d. DEG, ZnSO_4_·7H_2_O, and Zn(OAc)_2_·2H_2_O were obtained from Sigma-Aldrich, Munich, Germany. Ethyl acetate, acetone, methanol, and tetrahydrofuran were acquired from Chemsol-VN.

Methylene diphenyl diisocyanate (MDI) was obtained from Dow (Guangzhou, China) (Voracor CE101; 31.0% NCO, viscosity of 210 mPas (25 °C), and density of 1.23 g/cm^3^ (25 °C)). Triphenyl phosphate (TPP) was purchased from Sigma-Aldrich.

### 2.2. Characterization Equipment 

Fourier transform infrared (FTIR) experiments were performed on a JASCO FT/IR-6600 (Tokyo, Japan) using a diamond crystal and an angle of incidence 45°. Proton (^1^H) nuclear magnetic resonance (NMR) spectra were recorded with a Bruker ARX-500 NMR (Karlsruhe, Germany) spectrometer operating at 500 MHz and with DMSO-d_6_ as the solvent.

The LC-MS analysis was performed by using an Agilent LC 1290 coupled to an Agilent Q-TOF G6545A (Santa Clara, CA, USA). Column: Agilent Poroshell 120 EC-C18 (4.6 × 150 mm i.d., 2.7 μm particle size, Santa Clara, CA, USA). The elution was performed at room temperature by using solvent A (0.1% aqueous formic acid) and solvent B (0.1% formic acid in methanol) at a flow rate of 0.400 mL/min and a linear gradient of A/B from 90/10 to 0/100 in 70 min. The mass spectrometric analysis was carried out on an ISQ single quadrupole MS interfaced with an Ultra gas chromatograph from Thermo scientific (analysis parameters: Gas temperature 250 °C, gas flow 10 L/min, Nebulizer 35 psig, Waltham, MA, USA). 

Viscosity was determined by a Brookfield DVEELVTJO viscometer (Middleborough, MA, USA) at 31 °C using an LV2-62 spindle at 100 rpm.

Differential scanning calorimetry (DSC, Columbus, OH, USA) was performed with a METTLER STARe SW 11.00 instrument. The samples were heated/or cooled at a rate of ±10 °C/min in a nitrogen atmosphere. Thermogravimetric analysis (TGA, New Castle, DE, USA) was carried out on a Q500 Universal V4.5A TA instrument, with heating from room temperature to 800 °C at a heating rate of 10 °C/min in a nitrogen atmosphere.

The compression strength of PU-DZS and PU-DZS-TPP_25_ samples was measured by using a Universal Testing Machine-Model AG-X plus (Shimadzu, Kyoto, Japan) and following ASTM D1621. Each sample was cut into 4 blocks with dimensions of 50 × 50 × 25 mm^3^. The compression strength parallel to the foam rise was measured at a loading rate of 2.5 mm/min. The reported values were the averages and standard deviations of the testing results of 4 blocks of the same sample.

The morphology of PU rigid foams was examined by a scanning electron microscope (FE-SEM, Hitachi S-4800, Tokyo, Japan) at a voltage of 1.0 kV. The specimens were sputter-coated with a conductive layer of platinum.

The fire retardant properties were determined by LOI, UL-94, and cone calorimeter tests. The LOI (Qualitest) was determined according to ASTM D2863 for 5 samples with dimensions of 130 × 10 × 10 mm^3^. UL-94 was evaluated according to the standard tests ASTM D3801-96 for vertical burning (UL-94 V) and ASTM D635-98 for horizontal burning (UL-94 HB) with test specimen bars of 127 × 13 × 10 mm^3^. The cone calorimeter (FTT-Fire Testing Technology, East Grinstead, West Sussex, UK) determination was according to ISO 5660-1. Samples with dimensions of 100 × 100 × 10 mm^3^ were wrapped in aluminum foil and exposed to an external heat flux of 50 kW/m^2^.

### 2.3. PET Glycolysis Reaction under Microware Irradiation

In a 250-mL Erlenmeyer flask was added 48.0 g (0.250 mol) of PET flakes, 66.3 g (0.626 mol) of DEG (DEG/PET molar ratio = 2.5/1), and 0.427 g (0.89 wt % of PET) of ZnSO_4_·7H_2_O. The flask was covered with a watch glass. The household microwave oven was operated at a fixed power of 250 W and at 5 min operation intervals, followed by a 5 min pause, so that the reaction mixture would not boil. The microwave irradiation was continued until all PET flakes were completely dissolved. The total operation time was 80 min. The reaction mixture was cooled to room temperature and left overnight. The solid catalyst appeared at the bottom of the flask and was separated by decantation and centrifugation.

The quantity of volatile material lost during the reaction under microwave irradiation was determined as 7.62 g or 6.64% of the total initial weight of the reaction mixture. The obtained liquid product was characterized by FTIR, NMR and LC-MS.

Another glycolysis reaction was performed by using Zn(OAc)_2_·2H_2_O (0.68 wt % of PET) in place of ZnSO_4_·7H_2_O (0.89 wt % of PET). The quantities of the two catalysts used were calculated so that the Zn^2+^ molar percent were the same (molecular weight of ZnSO_4_·7H_2_O/Zn(OAc)_2_·2H_2_O = 287.53/219.50 = 0.89%/0.68% = 1.31).

### 2.4. PUF Synthesis by Reaction of Oligodiol with MDI

TPP (16.5 g, 25 pph of oligodiol) was heated to melting (approximately 60 °C) and then mixed well with 66.0 g (0.139 mol) oligodiol. The mixture was heated at 60 °C for 2 h under vacuum, and a homogeneous mixture was obtained. The liquid mixture was combined with 2.64 g silicone, 0.66 g purified water, 8 drops of dibutyltin dilaurate (DBTDL) and 8 drops of triethylamine (TEA). MDI (85.8 g, 135.5 g/eq) was added to the above oligodiol mixture and mixed well by using a mechanical stirrer for 8 s. The mixture was quickly transferred to a carboard mold (8 × 13 × 14 cm^3^) and allowed to rise freely. The quantity of TPP used was equivalent to 10% of the total PU weight. Other PUF samples with lower TPP contents were also prepared.

PU-DZS and PU-DZA samples, without added TPP, were also made by the same procedure, except that 79.0 g MDI was used.

## 3. Results and Discussion

PET glycolysis was performed by using DEG/ZnSO_4_·7H_2_O under microwave irradiation. The transesterification of PET normally requires a large excess of diol compared to PET to wet the solid PET with liquid diol and to shift the equilibrium to lower molecular weight oligomer products or a liquid oligodiol. However, a large excess of diol reactant should be avoided, because it could create other waste material during purification. Furthermore, the properties of the oligodiol mainly depend on the excess diol reactant, when not removed from the product mixture. Therefore, a minimum ratio diol/PET must be used. When the DEG/PET molar ratio was reduced to 2.0/1.0, immediately after the reaction at high temperature, the product was a liquid or the PET flake was completely converted into liquid oligodiol. However, after cooling and leaving at room temperature for 24 h, the product became opaque, i.e., some small solid particles appeared, or the molecular weight of the obtained oligomer was high and insoluble in the reaction mixture at room temperature.

When the DEG/PET molar ratio was increased to 2.5/1.0, after long storage at room temperature, the product still existed as a viscous liquid. Therefore, the minimum DEG/PET molar ratio should be 2.5/1.0. This ratio is smaller and better than the value of 4/1, as reported by Roy et al. [14] when they used Zn(OAc)_2_·2H_2_O (0.5 wt% of PET) under microwave irradiation at 450 W.

### 3.1. Characterization of the Oligodiol Products

The oligodiol obtained from the reaction of DEG and PET catalyzed by ZnSO_4_·7H_2_O (after removal of the catalyst) was analyzed by FTIR, NMR, and LC-MS.

**Figure 1:**
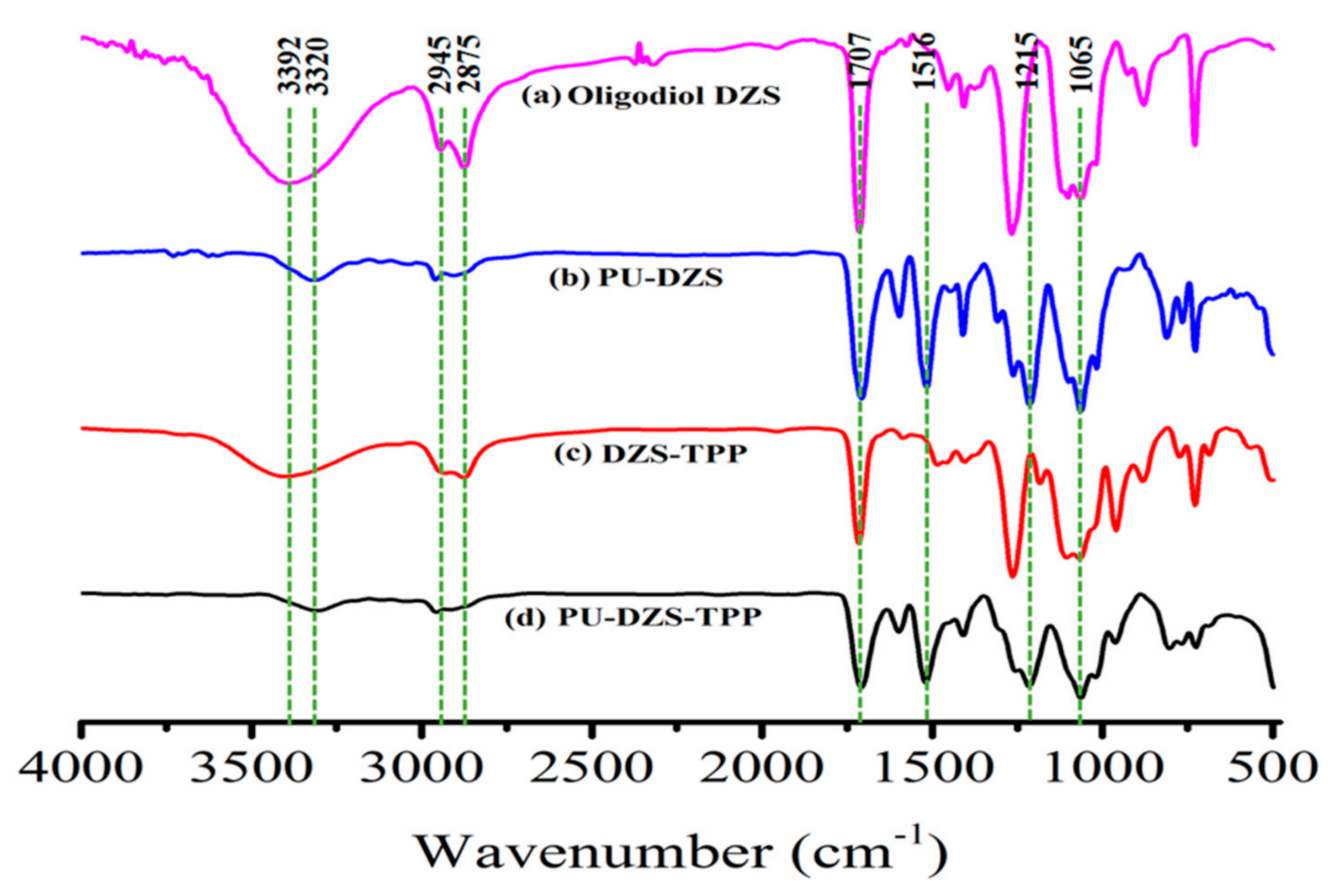
FTIR-ATR spectra of (**a**) oligodiol DZS; (**b**) polyurethane prepared from DZS and MDI; (**c**) a mixture of DZS and TPP (25 pph); and (**d**) polyurethane prepared from DZS, TPP and MDI.

FTIR-ATR spectra of the oligodiols prepared by using ZnSO_4_·7H_2_O, shortened as DZS (Figure 1), and Zn(OAc)_2_·2H_2_O, shortened as DZA, showed almost the same features. Compared to the FTIR spectrum of PET, the presence of the –OH end groups of the oligodiol was confirmed by a broad band at 3392 cm^−1^, while the ethylene ether moiety appeared as bands at 2945 cm^−1^ for the CH_2_ asymmetric stretching, 2875 cm^−1^ for the CH_2_ symmetric stretching, and 1065 cm^−1^ for the C–O stretching.

The DMSO-d_6_ solution containing oligodiol was treated with D_2_O to transform all the O–H end groups of oligodiol to O–D so that it would not appear in the spectrum and it would not interfere with the quantitative integral measurement. The strong signal at 3.64 ppm was due to HOD.

**Figure 2:**
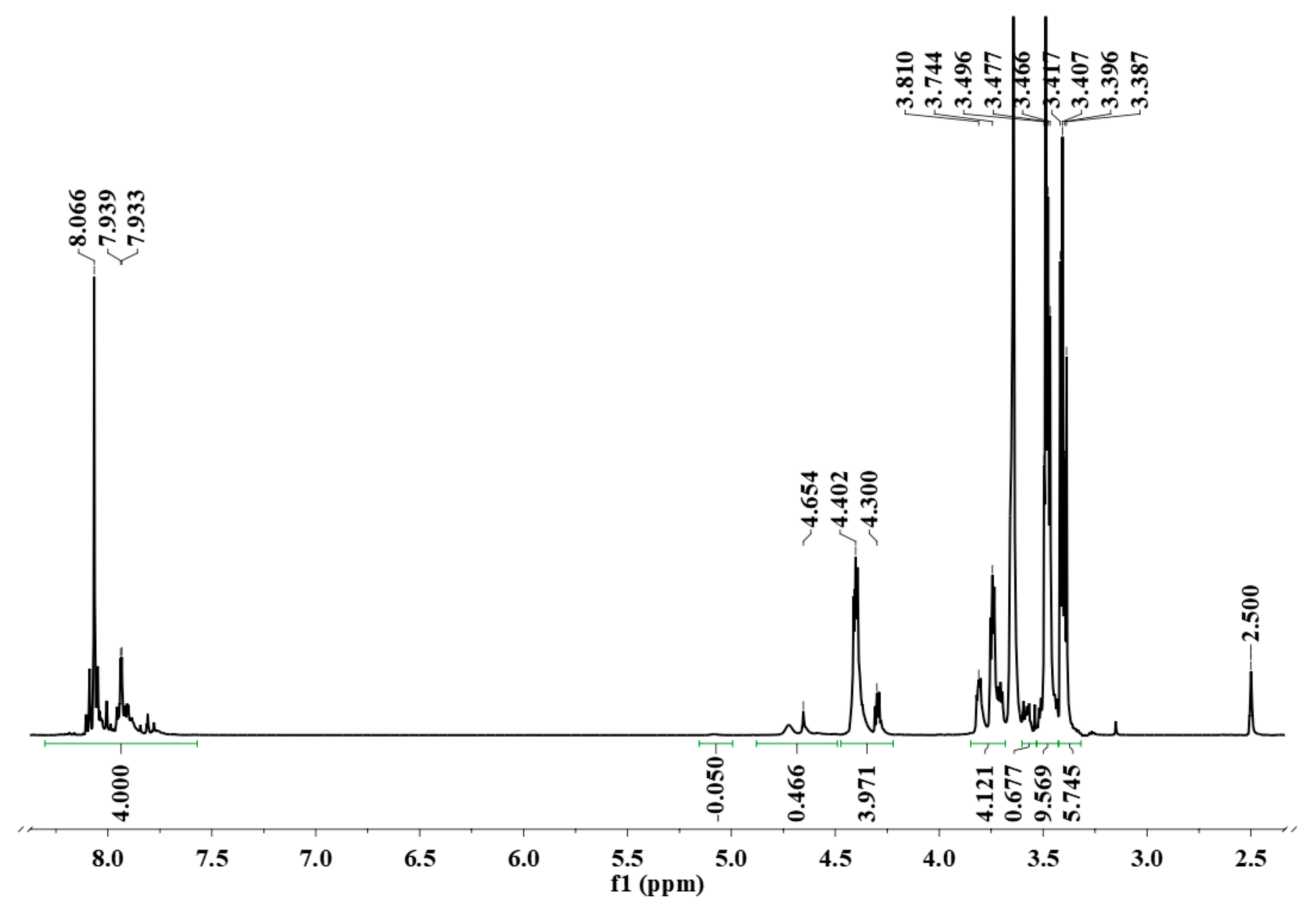
^1^H-NMR spectrum (DMSO-d_6_-D_2_O) of DZS oligodiol.

The ^1^H-NMR spectrum (Figure 2) shows aromatic protons from 7.75 to 8.15 ppm, and aliphatic CH_2_ appears as multiplet signals from 3.75 to 4.50 ppm. Weak signals at 3.58, 4.75 and 5.10 ppm are due to impurities. Resonance signals at 4.3 and 4.4 ppm are typical for –C**H**_2_OCOAr with an integral of 3.97 protons, almost equal to 4.00 protons of aromatic CH. The ^1^H-NMR integrals are normalized with respect to one terephthalate unit or 4 aromatic protons. Resonances at 3.74 and 3.80 ppm are due to –C**H**_2_CH_2_OCOAr with 4.12 protons that also close to 4.00 protons. The –C**H**_2_OC**H**_2_– of ethers or –C**H**_2_OH of end groups appearing at 3.41 and 3.49 ppm contributes to 15.31 protons.

The total number of aliphatic protons is 23.41. One monoethylene glycol unit, abbreviated as M, has 4 protons; therefore, the number of the M unit must be 5.85. The total aromatic protons are 4.00 (from the spectrum). One terephthalate unit, abbreviated as T, has 4 protons; therefore, the number of T units must be 1.00. Consequently, from the ^1^H-NMR spectrum, the molar ratio M/T must be 5.85/1.00. The initial D/(TM) molar ratio equals 2.5 (D stands for a DEG unit, which is equivalent to 2 M units, and PET is represented by TM) or the initial M/T molar ratio equals 6/1. A small difference of 0.148 M between the initial and final ratios could be due to the decomposition of M or D to form volatile materials during the reaction at high temperature under microwave irradiation. The volatile materials were isolated and characterized by GC-MS as a mixture of water and the decomposition products of M and D, such as 1,4-dioxane, acetaldehyde, 2-methyl-1,3-dioxolane, etc. [18].

From the ^1^H-NMR spectrum, we could also determine the molar ratio of M (ether -C**H**_2_OC**H**_2_)/M (ester –C**H**_2_OCO) = 19.44/3.97 = 4.90/1. The newly formed ether bond must be [(4.90/2) − 1]/1 = 1.45/1, i.e., 1.45 mol ether bonds were formed per 1 ester unit. In theory, for 1 mol of PET or (TM) units completely reacted with 2.5 mol of D via the transesterification and ether formation, the following chemical equations were proposed:

(1) Transesterification: (TM) + D → DTM
(2) Ether formation: DTM + 1.5D → D_2.5_TM or equivalent to TM_6_.

The experimental value of 1.45 mol new ether bonds was quite close to the 1.5 mol proposed by the above calculation and theoretical equations.

The mass reduction during the reaction came not only from the decomposition of M or D but also the ether formation (2), when water was lost. The percent of mass reduction due to water removal was 1.45 × 18 × 100%/(2.5 × 106 + 192) = 5.70% (only 1.45 mol of new ether units was formed, and the other ether units came from the D structure). The percent of M units lost during the reaction was 0.148 × 62 × 100%/(2.5 × 106 + 192) = 2.01%. The total mass loss calculated from the ^1^H-NMR spectrum was 7.71%. The percent of experimental loss was 6.64%. The two values were very close, therefore, the characterization by ^1^H-NMR was correct.

Finally, the ^1^H-NMR spectrum demonstrated that both the transesterification of the diol with terephthalate ester and the ether formation from the alcohol end groups of DEG occurred during the glycolysis of PET by DEG. Consequently, the obtained product was a complex mixture of novel oligo-ester-ether-diols, and the quantity of excess DEG reactant was negligible.

The oligodiol sample was separated at first by liquid chromatography (LC), and each fraction at a different retention time was further analyzed by mass spectrometry (MS). From the mass spectrum, the molecular weight of each component was determined, and the molecular formula/structure was proposed.

The complex oligodiol structures were described by using abbreviated characters, such as T for the terephthalate unit or [COC_6_H_4_COO], M for the monoethylene glycol unit or [CH_2_CH_2_O], and D for the diethylene glycol unit that equals M_2_. Combining different T, M and D units and one molecule of water resulted in various oligodiol structures and molecular weights. The proposed structure would be correct if the calculated molecular weight was almost equal to the experimental m/z value from MS.

**Figure 3:**
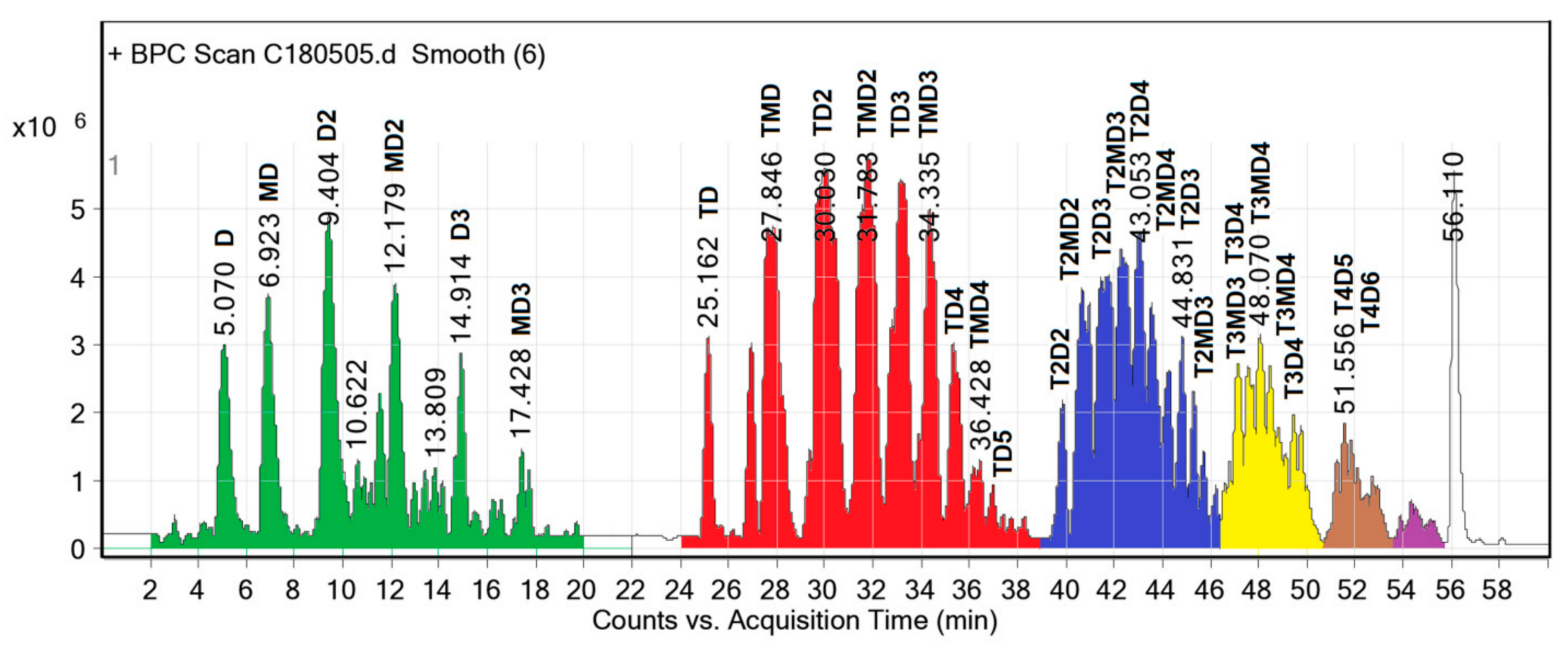
Total ion current chromatogram from the LC experiment of the oligodiol DZS sample.

The chromatogram (Figure 3) was separated into five groups, corresponding to five homologous series of structures or 67 different individual structures. Extremely weak signals were not considered.

(1) Retention time from 0 to 20 min.

Figure 3 shows a separation of the major components from 0 to 20 min, including D, MD, D_2_, MD_2_, D_3_ and MD_3_ as a homologous oligoether series of ethylene glycol (M) and diethylene glycol (D). The increment of homologous series was one M unit. The higher molecular weight molecules required longer retention time on the column. The most abundant component in this homologous oligoether series was the D_2_ that was formed from two D molecules. The longest chain of this series was MD_3_ that was created from at least three new ether bonds among one M and three D units, corresponding to an initial DEG/PET molar ratio of 2.5/1. This homologous oligoether series was not detected in the LC-MS result of the sample obtained after treatment with an aqueous 20% NaCl solution [18], because the components had a high solubility, even in brine solution, and were removed from the final product mixture.

The reactant D not only contributes to the homologous oligoether series, but its peak height in LC (Figure 3) also follows a good normal distribution curve. Here, we can conclude that the quantity of D is not in excess in the product mixture, but in an equilibrium quantity from the transesterification and ether formation reactions. For this reason, the removal of D from the reaction mixture is almost impossible and unnecessary.

(2) The retention time from 22 to 39 min in the chromatogram displayed peaks of a homologous monoterephthalate series with structures of increasing molecular, namely, TD, TMD, TD_2_, TMD_2_, TD_3_, TMD_3_, TD_4_, TMD_4_ and TD_5_, with the most abundant molecules being TD_2_ and TMD_2_. The increment of this homologous series was also one M unit. The smallest monoterephthalate structure was TD, which should be rewritten as MTM due to the probability of a carboxylic acid end group in TD being very low when diol was used in excess. The highest molecular weight component of the monoterephthalate series was TD_5_, arranged in the order of DTDDD or DDTDD, and formed by two ester and two ether groups.

Analogously, the diterephthalate series appeared from 39–46 min, the triterephthalate series eluted from 46–51 min, and the retention time of the tetraterephthalate series was from 51–53 min. All individual structures were also confirmed by MS.

Based on the above LC analysis, we can conclude that the oligodiols obtained without the aqueous 20% NaCl solution treatment are a complex mixture due to the presence of oligomers with longer ether chains, namely, TD_4_, TMD_4_, TD_5_, T_2_D_4_, etc., or that this is a mixture of various oligo-ester-ether-diols. Once again, the transesterification and ether formation reactions during PET glycolysis by DEG are confirmed by LC analysis. This is the first time that the structures of oligo-ester-ether-diols are experimentally confirmed. Here, we refer to the glycolyzed product as an oligo-ester-ether-diol and not as the commonly used polyol because the term polyol could be misunderstood as a polymer or a monomer that has multiple alcohol groups. Our product is an oligo-ester-ether-diol that has a low molecular weight with two alcohol end groups and contains terephthalate ester and ethylene glycol ether moieties in the linear chain. This novel diol is expected to offer the advantages of both ester and ether diols in the polyurethane formulation.

**Figure 4:**
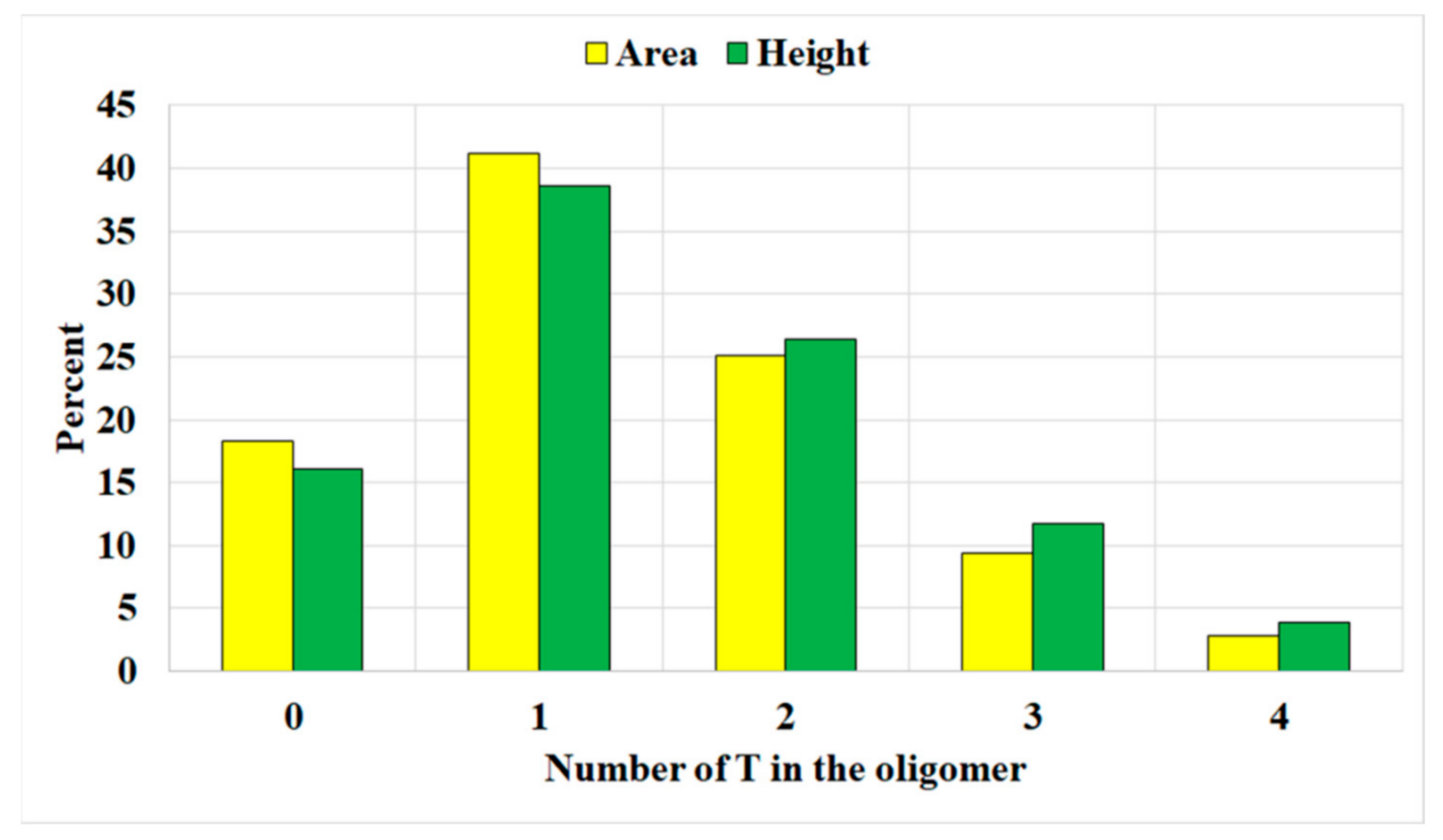
Distribution of terephthalate unit numbers in the molecule as determined by the area or height of the peaks in the total ion current chromatogram.

The distribution of terephthalate unit numbers in the molecule is depicted in Figure 4. The major component of the oligodiol is monoterephthalate. The oligoethers without terephthalate units contribute to approximately 17%. The components containing larger terephthalate unit numbers have lower abundances. From the distribution, the average terephthalate unit in one oligomer chain is calculated as 1.367 by peak area or 1.492 by peak height.

The quantitative analysis of the LC-MS also shows different responses of peak areas and heights. At the lower terephthalate unit number or lower molecular weight side (Figure 4), the intensities based on the area are stronger than those based on height. In contrast, at higher molecular weight regions, the relationship is reversed. For most LC analyses, the peak areas are used for quantitative calculations.

G.P. Karayannidis et al. [8] used gel permeation chromatography to characterize the PET glycolysis product with DEG by using a 2.2/1 molar ratio with conventional heating. Three peaks in the GPC chromatogram were identified as three oligoester diols with mono-, di- and tri-terephthalate units. In our research project, oligodiol was characterized by the LC-MS method, and up to 67 different structures of 5 homologous oligo-ester-ether-diol series containing from zero to four terephthalate units were confirmed.

The calculations based on the area ratios of peaks in the chromatogram resulted in M_n_ = 475 g/mol, M_w_ = 581 g/mol, PDI = 1.22, the average molecular formula T_1.37_M_5.79_, and a molar ratio of M/T = 4.23. When the height ratios of peaks in the chromatogram were considered, it resulted in M_n_ = 507 g/mol, M_w_ = 615 g/mol, PDI = 1.21, the average molecular formula T_1.49_M_6.08_, and a molar ratio of M/T = 4.08.

The number average molecular weight (M_n_) and weight average molecular weight (M_w_) are calculated by using the formulas $M_{n}=\frac{\sum^{\text{​}}N_{i}M_{i}}{\sum^{\text{​}}N_{i}}$ and $M_{w}=\frac{\sum^{\text{​}}N_{i}M_{i}^{2}}{\sum^{\text{​}}N_{i}M_{i}}$, where N_i_ is the relative area or relative height of the peak in the chromatogram, and M_i_ is the molecular weight, as determined by mass spectrometry.

The M/T ratio calculated from LC was substantially smaller than the value obtained from ^1^H-NMR (5.825). We found that the area ratios of LC peaks matched the ^1^H-NMR spectrum better, and we chose M_n_ = 475 g/mol as the molecular weight of the oligodiol.

In comparison, even though the PET glycolysis was conducted in a microwave with a higher DEG/PET molar ratio of 4/1 and in the presence of Zn(OAc)_2_, a larger M_n_ of 965 was obtained [13].

Even though DEG was used in excess, the LC analysis showed that the quantity of unreacted DEG reactant in the oligodiol product was small due to the transesterification and ether formation occurring simultaneously and reaching an equilibrium state to form the oligo-ester-ether-diol. For this reason, purification by treatment with a cool 20% NaCl solution [18] was unnecessary. In this research project, we use the glycolyzed product mixture directly for PU synthesis without purification steps, therefore, no additional waste is created, and a larger quantity of diol from waste PET is used.

The viscosity of the liquid oligodiols was determined by a Brookfield DVEELVTJO viscometer at 31 °C using an LV2-62 spindle. The viscosity value of the DZA sample was 219 cp and that of the DZS sample was 268 cp. The DZS oligodiol prepared by using the ZnSO_4_·7H_2_O catalyst showed a higher viscosity and thus a higher molecular weight than that of the DZA oligodiol. Both oligodiol samples remained in the liquid state at a temperature as low as −15 °C.

### 3.2. Recovery and Reuse of the ZnSO_4_·7H_2_O Catalyst

The FTIR spectrum of the ZnSO_4_·7H_2_O catalyst recovered from the glycolysis reaction of PET with DEG and showed most typical peaks of the initial commercial ZnSO_4_·7H_2_O. This recovered ZnSO_4_·7H_2_O was reused as a catalyst for other PET glycolysis reaction batches and showed no change in reactivity. Therefore, ZnSO_4_·7H_2_O is not only a good catalyst to prepare light-color liquid diol from PET but also a good catalyst that is heterogeneous at room temperature, and consequently, it is readily removed from the reaction mixture and reused.

### 3.3. Polyurethane Characterization

**Scheme 1:**
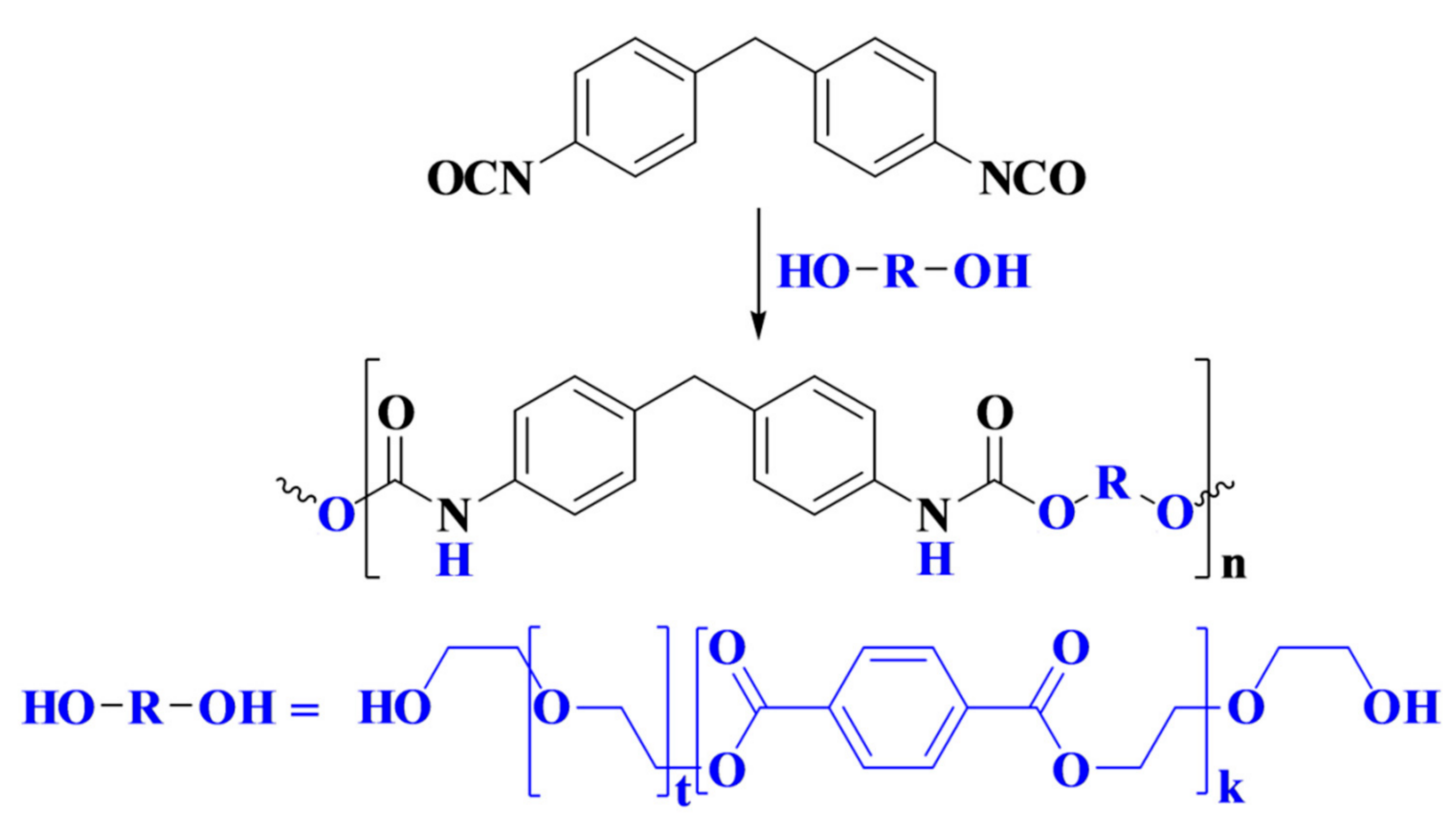
Polyurethane synthesis from oligodiol (k = 0–4, t = 0–6) and MDI.

Rigid polyurethane foam (PUF) was prepared by the reaction of oligo-ester-ether-diol with methylene diphenyl diisocyanate (MDI), as depicted in Scheme 1.

Foam was formed by the reaction of isocyanate with water to form carbamic acid, which is an unstable intermediate that is readily decomposed to evolve carbon dioxide and generate an amine. Consequently, this amine further reacted with isocyanate to form urea. For this reason, MDI was used in excess, and NCO/OH ratio was normally larger than 1.

At first, PU samples were prepared without adding the flame retardant so that the best foaming formulation and conditions were discovered. After that, by using the same formulation and procedure, the oligodiol samples were treated with triphenyl phosphate (TPP), as a flame retardant, before the foaming process.

The ATR-FTIR spectra of PUs prepared from oligodiols DZS and DZA resulted in almost the same features. The spectra (Figure 1b,d) showed typical peaks of the urethane group (–NHCOO–) at 3320 cm^−1^ for NH stretching, 1707 cm^−1^ for C=O stretching, 1516 cm^−1^ for N–H bending and 1215 cm^−1^ for C–O stretching. The C=O stretching at 1720 cm^−1^ for the ester in the diol DZS reactant was shifted to a lower wavenumber for the C=O of urethane. The band at 1597 cm^−1^ was caused by the C=C stretching of benzene rings. Additionally, the ether C–O stretching resulted in strong bands at 1099, 1065, and 1018 cm^−1^.

After mixing TPP with DZS, the FTIR spectrum (Figure 1c,d) showed a weak absorption at 1184 cm^−1^ and a medium peak at 960 cm^−1^ for P–O stretching vibrations.

#### 3.3.1. Density, Pore Size, and Compression Strength

The measured densities of PU-DZS and PU-DZA were almost the same (102 ± 10 kg/m^3^). Their values depended only on the quantity of water used as a foaming agent, and not that of the oligodiol. An attempt to reduce the density of the foam by increasing the amount of water in the formulation was not successful. The foam formed quickly, and collapsed. The PUF prepared from copolymer or polyester polyols usually has a density in the range of 25 kg/m^3^ to 100 kg/m^3^ [19].

When TPP was added, the density of the PUF became lower (90 kg/m^3^) or the foaming process was more successful. TPP could play roles of a good solvent and a compatibilizer for oligodiol and MDI.

**Figure 5:**
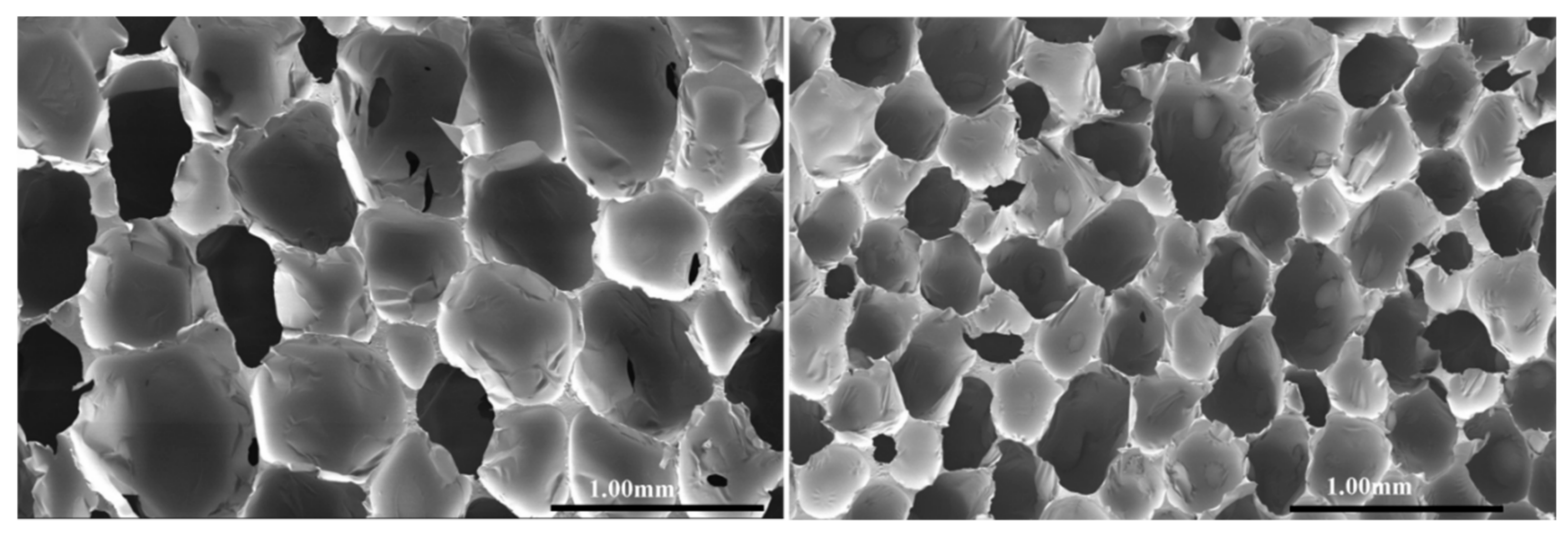
SEM micrographs of PU-DZS (**left**) and PU-DZS-TPP_25_ (**right**).

Both samples showed quite uniform cells (Figure 5). The average cell size of PU-DZS (left) is larger than that of PU-DZS-TPP_25_ (right), with the average diameters being 619 ± 65 μm and 408 ± 39 μm.

**Figure 6:**
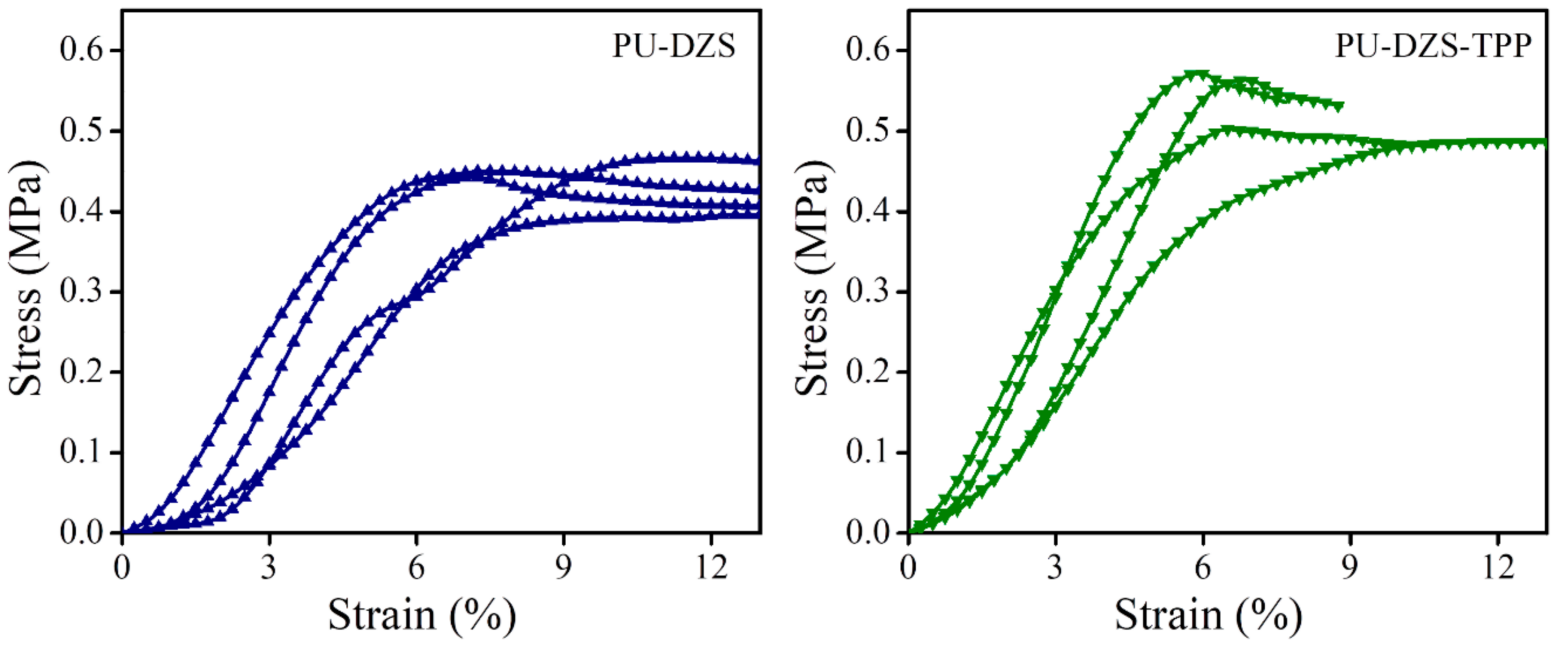
Compression stress-strain curves of PU-DZS (**left**) and PU-DZS-TPP_25_ (**right**) samples.

Compression strength was evaluated according to ASTM D1621 with a speed of 2.5 mm/m (Figure 6). The smaller pore size of the PU-DZS-TPP_25_ sample enhanced its yield strength (530 ± 45 kPa) compared to that of the PU-DZS sample without TPP (433 ± 31 kPa). The strain at the yield point showed a reverse relationship with corresponding values of 7.21 ± 1.90 and 9.4 ± 2.1%. When TPP was added, the cell size of the PUF decreased; therefore, it can withstand a stronger force, i.e., a higher yield stress and lower yield strain. Even though TPP reduced the T_g_ of the PU-DZS sample, compression tests were carried out at room temperature, which is below the T_g_; therefore, the compression strength did not depend on T_g_.

The density of our PUF samples (90–102 kg/m^3^) is lower than the similar PUF prepared from PET glycolized by EG (from 150 to 510 kg/m^3^ [16]) and the compression strength of our PUF samples is also higher (433–530 kPa compared with 140–185 kPa [16]). These better results could be explained by the effective role of flexible liquid oligo-ester-ether-diol prepared by PET glycolysis with DEG in foam formation and in mechanical strength enhancement.

#### 3.3.2. Thermal Properties

The thermal transitions of the PUF samples were detected by DSC. The samples were heated (a) from 25 to 120 °C, (b) cooled from 120 to −50 °C and (c) reheated from −50 to 200 °C with heating/cooling rate ±10 °C/min.

**Figure 7:**
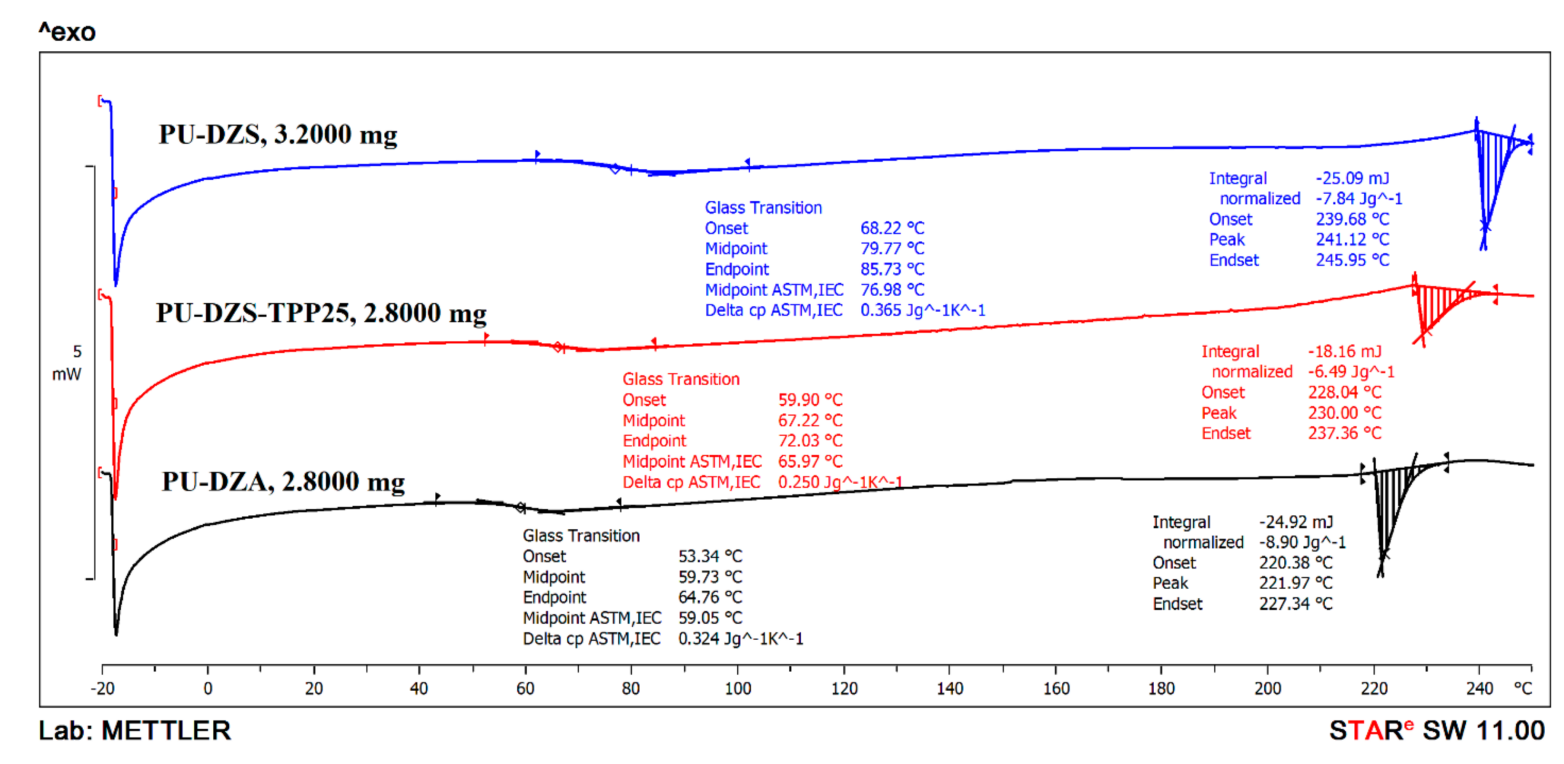
DSC curves of the PUF samples prepared from MDI and diols: DZS, DZS-TPP_25_ and DZA. The second heating from −50 to 200 °C at a rate of 10 °C/min.

The glass transition temperature of the PUF is typical for ester or ether oligodiol soft segments in the chain. The DSC curves (Figure 7) show that PU-DZS (T_g_ = 79.77 °C) has a higher glass transition temperature than that of PU-DZA (T_g_ = 59.73 °C). This result could come from the fact that PU-DZS was prepared from an oligodiol that has a higher molecular weight than that of PU-DZA, as confirmed by the higher viscosity. As TPP was added to PU-DZS, the T_g_ reduced to 67.22 °C (PU-DZS-TPP_25_ curve). This is good evidence for the role of TPP as a plasticizer. The small TPP molecule was very compatible with the oligodiol soft segments in the PUF, and TPP also enhanced the flexibility of the soft segments; therefore, it reduced the T_g_ of the PUF.

At the high temperature range, the endothermic peaks that are typical for a melting process have the same increasing trend as that of the T_g_ values. The PUF has a high T_g_ and a high T_m_. These can be the melting points of the soft segments of the terephthalate crystalline structure. The contribution of ethylene terephthalate segments to the total mass of PU in the PU-DZS sample is calculated as 48g/(48 + 66.3 − 7.62) × 100% = 45.0% of the oligodiol. The oligodiol was used as 66/145 × 100% = 45.5% of the PUF. The mass percent of ethylene terephthalate units in the PUF is 45.0% × 45.5% = 20.5%. This is also the mass percentage of the PET waste contribution in the PUF. Therefore, the % crystallinity of ethylene terephthalate units is (7.84(J/g)/140(j/g)/0.205) × 100% = 27.3%.

**Figure 8:**
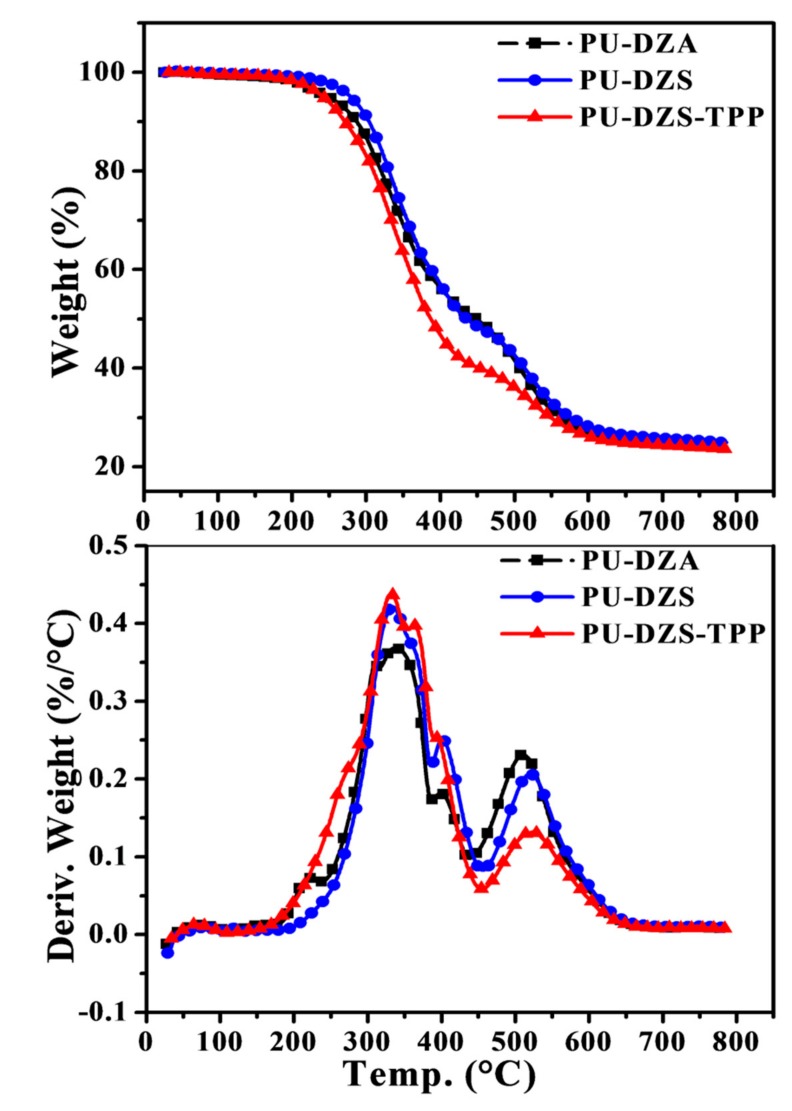
TG (**up**) and DTG (**bottom**) curves of PU-DZS, PU-DZA, and PU-DZS-TPP_25_ samples at a heating rate of 10 °C/min in N_2_.

Two main degradation stages were distinguished following the TG and DTG curves (Figure 8). When heating the PU-DZS sample from room temperature to 200 °C, there is a small weight loss due to volatile material (0.45%). A maximum weight loss is observed at 334 °C for the first stage from 200 °C to 460 °C, with an overall weight loss of 52.1%. In this temperature range, B.H. Kim, K. Yoon, and D.C. Moon [20] suggested that the sequence of thermal degradation of rigid and soft polyurethanes based on methylene diphenyl diisocyanate was from hard to soft segments.

The second stage of decomposition covers 460 °C to 650 °C, with a maximum peak at 520 °C. The onset of the first decomposition at 284 °C is higher than the reported value of 260 °C [13].

The low thermal decomposition of PU-DZA at 220 °C in the DTG curve, compared to that of PU-DZS, could be explained by the presence of Zn^2+^ catalyst residue and impurities in DZA, as evidenced by the dark color of the oligodiol. From room temperature to 438 °C, the PU-DZA sample lost 41.50%.

TPP is a flame retardant, and it has simultaneously worked as an ecologically friendly plasticizer for triacetyl cellulose [21] and poly(vinyl alcohol) [22]. When TPP was added to the PUF, the decomposition trends were almost unchanged. The char content at 800 °C of PU-DZS-TPP_25_ (23.65%) was not quite different from that of the PU-DZS sample (24.92%). The calculated residue at 800 °C of PU-DZS-TPP_25_ was 0.90 × 24.92% + 0.10 × 0.00% = 22.43%. Assuming that the char was only formed from TPP, we had equation 0.90 × 24.92% + 0.10 × X% = 23.65%. The solution to this equation is X = 12.2%. Consequently, at least 87.8% of the TPP converted into the gaseous phase and only 12.2% converted into char, or TPP mainly played the role of flame retardant in the gaseous phase. This conclusion again confirmed our previous statements [22].

**Figure 9:**
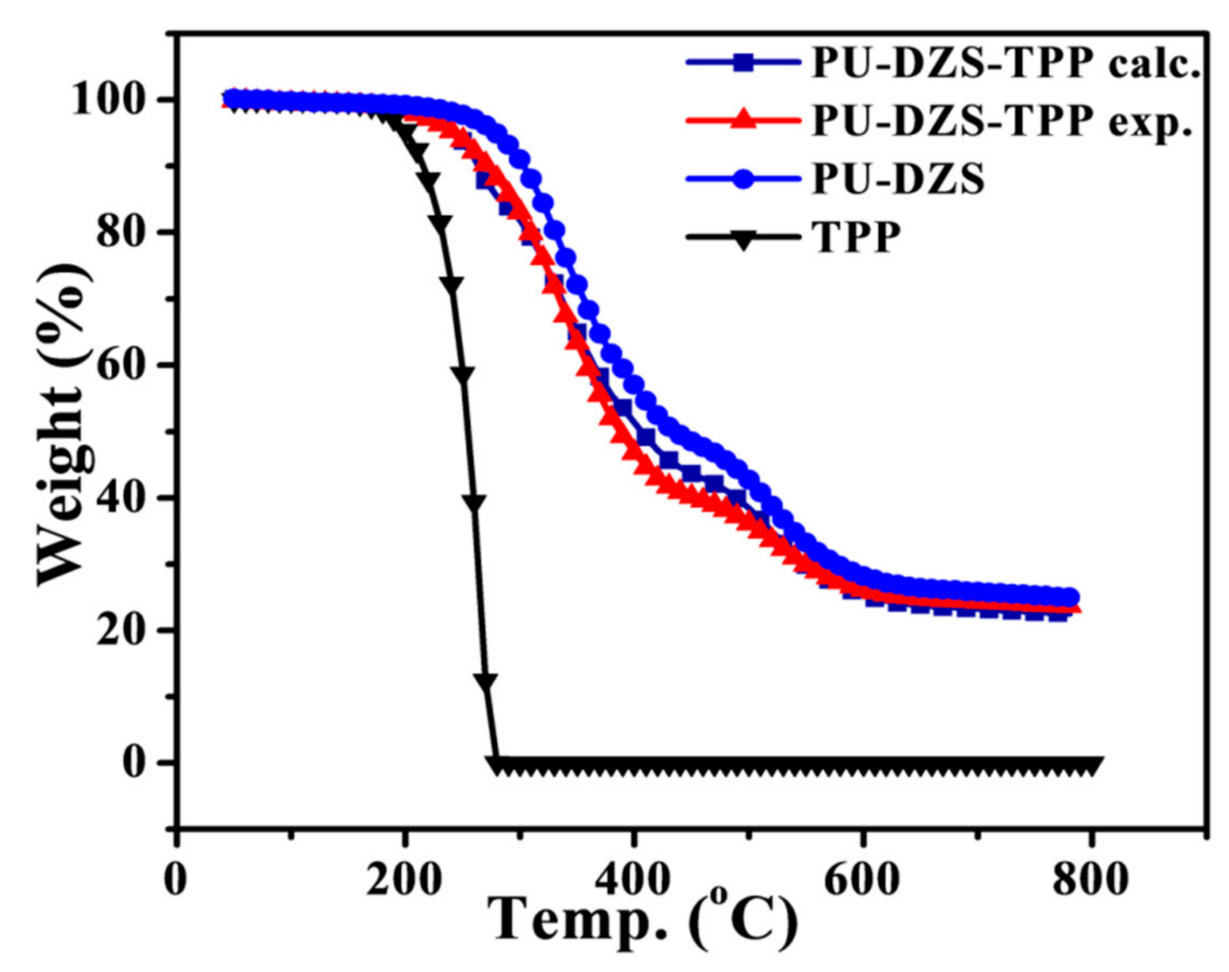
TGA thermograms of TPP, PU-DZS, and their mixtures (experimental and calculated) in N_2_ at a rate of 10 °C/min.

To provide proof of the TPP effect on the thermal degradation behaviors of the PUF, experimental TGA data were compared with TGA values calculated by using the additive rule (Figure 9). The obtained results show that the addition of TPP slightly accelerated the thermal decomposition of PU-DZS in the temperature range of 320–530 °C. There was an interaction between TPP and PU-DZS, but it did not significantly contribute to the fire retardant property. The main flame retardant mechanism for TPP occurred entirely via the gas phase, and the char layer of PU-DZS, acting in the condensed phase, was partially responsible for the flame retardancy of the mixture.

#### 3.3.3. Flammability and the Mode of Action of PUF

Rigid PUF is a highly flammable material, and it is not easy to find an effective single-component halogen-free flame retardant (FR). To achieve good flame retardancy, the addition of a char-forming agent along with a phosphorus FR is required. However, in this study, the rigid PUF prepared from recycled PET oligodiol contains aromatic moieties and is more thermally stable. Therefore, a single phosphorus FR (as TPP) was added in 5–25 pph loadings to find the UL94 V-0 ranking. The designated compositions and test results are shown in Table 1. The PU-DZS sample without TPP was highly combustible, having a low LOI value of 17%; the flame propagated along the test specimen to the holding clamp with a horizontal burning rate of 120 mm/min, so no rating was recorded for the UL94 HB test. When 5 pph TPP was added, the PUF sample also did not satisfy the UL-94 HB test; however, the horizontal burning rate decreased to 75 mm/min. No UL-94 V rating was recorded for the PU-DZS-TPP_5_ sample. The sample with the 15 pph TPP loading obtained the V-1 rating, while the V-0 ranking was obtained for the PUF sample containing 25 pph TPP; furthermore, the LOI value was found to be significantly increased from 17% to 21%.

The UL-94 and LOI results are quite good and surprising when considering the rather low content of phosphorous in TPP. Commonly, additions of more than 30 wt % TPP to polymers can achieve good flame retardancy. The results observed in this study—that only 25 pph (10 wt %) TPP loading could give a UL-94 V-0—clearly indicate that the chemical structure of the FR or the polymer itself and the fireproof mode of action of the FR and/or the polymer matrix need to be considered.

It is well known that TPP vaporizes and yields active radicals such as PO_2_^●^, PO^●^, and HPO^●^ during combustion. These radicals act as scavengers for H^●^ and OH^●^ radicals and thus extinguish the flame of the combustion process [23,24]. As mentioned above, the PU-DZS prepared from recycled PET oligodiol and MDI has aromatic moieties and becomes more thermally stable. According to the thermal decomposition in Figure 8, the weight loss of PU-DZS is 74% in the temperature range of 250–650 °C, leaving 26% residual char. This means that the flame retardancy mechanism of PUF mainly depends on the gas phase, but the condensed phase also plays an important role. TPP evaporated in an early step and acted as a radical scavenger or crosslinker for the pyrolytic products of PU-DZS.

**Figure 10:**
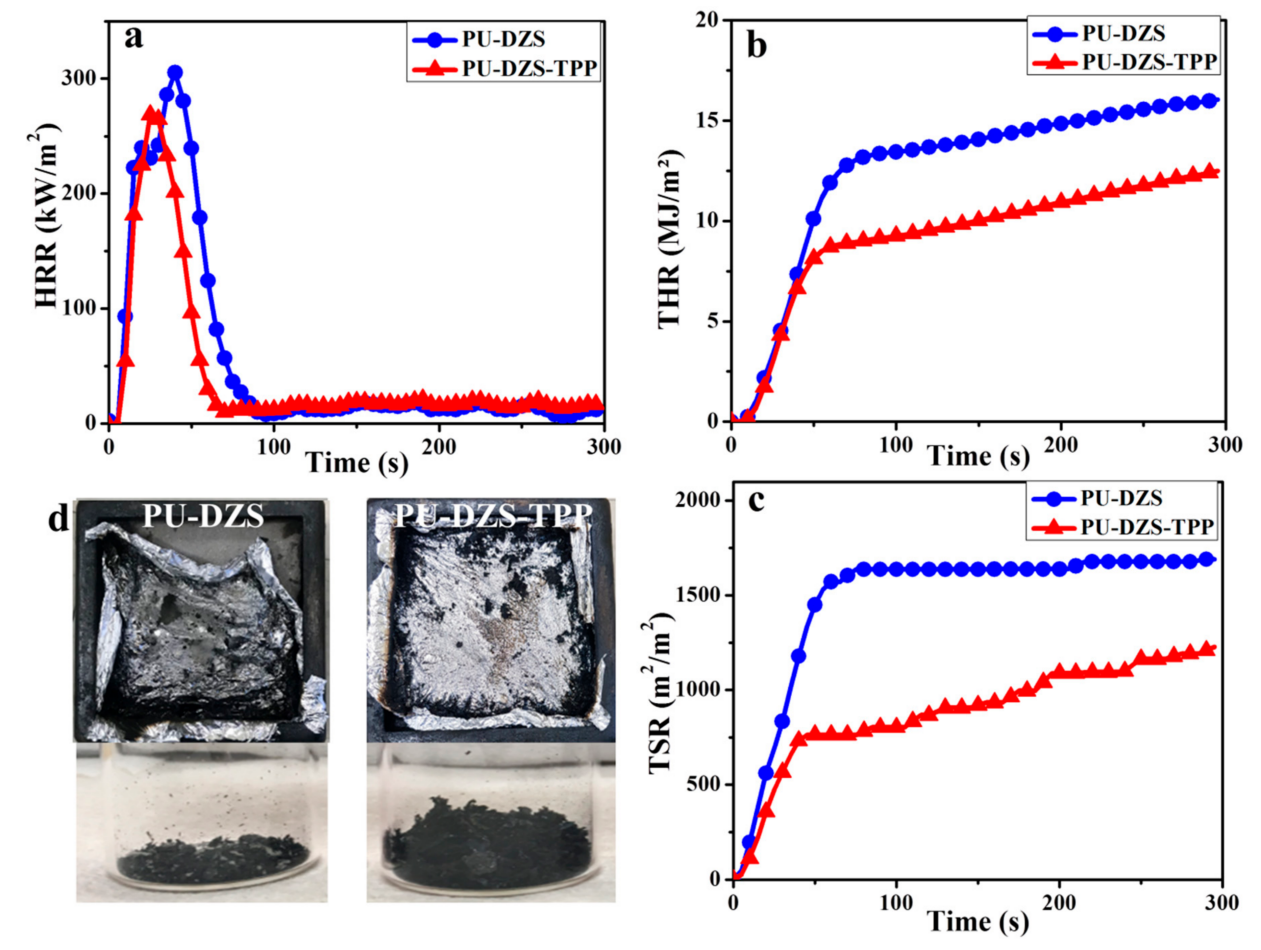
(**a**) HRR; (**b**) THR; and (**c**) TSR data and (**d**) the digital photos of the residual char after cone calorimeter tests of PU-DZS and PU-DZS-TPP_25_.

The cone calorimeter is a bench-scale instrument used to determine the flammability parameters of materials in laboratory. It is one of the most useful tests to compare with real-world fire conditions, and there were correlations between the cone calorimeter data and UL-94 V rankings [25,26,27]. The flammable performance of the neat PU-DZS and PU-DZS-TPP_25_ was evaluated by the cone calorimeter, and the testing results including the heat release rate (HRR), the peak HRR (PHRR), the total heat release (THR) and the total smoke release (TSR) are shown in Figure 10. The UL94 and LOI results are in good agreement with the cone calorimeter data. One might expect to observe lower PHRR and THR values for PU-DZS-TPP with a better UL-94 V ranking. However, PU-DZS-TPP_25_, classified as UL94 V-0, had lower PHRR, THR, and TSR values than the neat PU-DZS (no UL94 rating).

**Figure 11:**
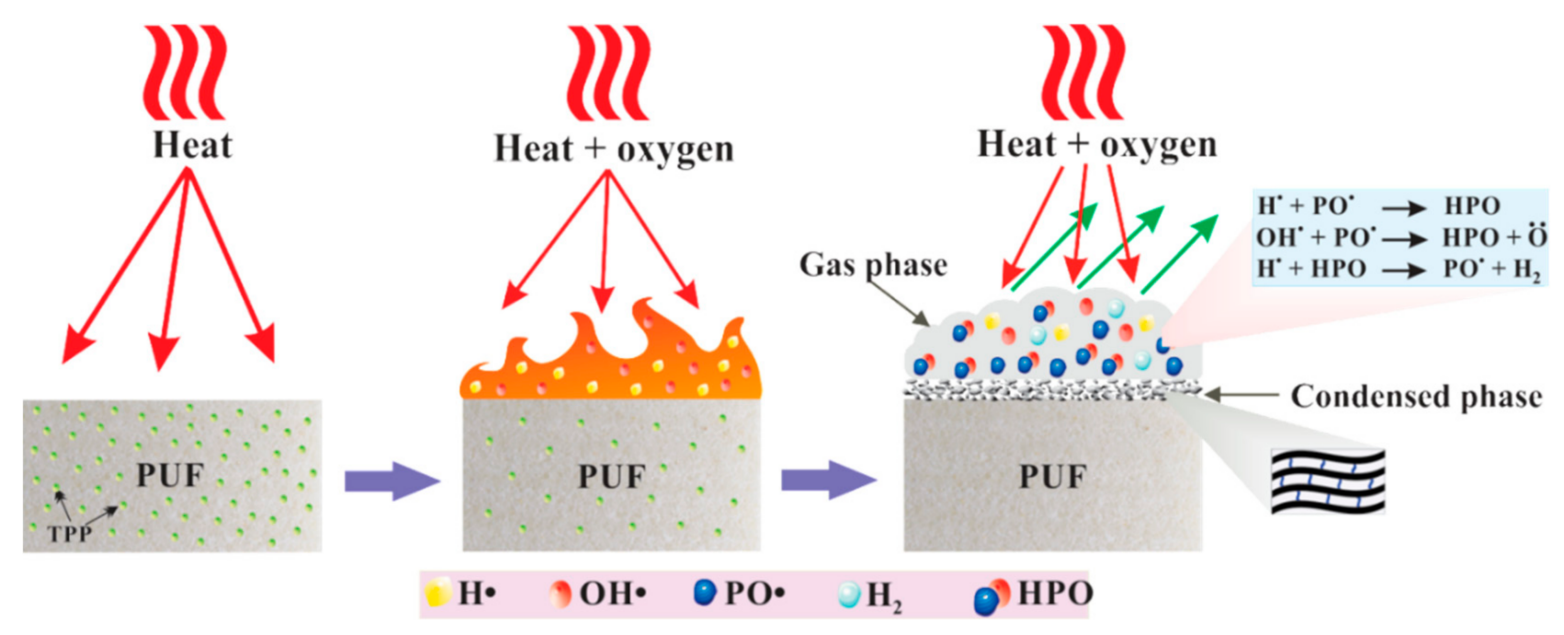
Schematic illustration of the flame-retardant mode of action of PU-DZS-TPP_25_.

The PHRR, THR, and TSR of PU-DZS-TPP_25_ were reduced by 12.9%, 22.8%, and 26.7%, respectively. As mentioned above, we know that the mode of action of TPP mainly inhibits gas-phase combustion, which effectively lowers the amount of oxygen consumed and the heat generated. In other words, TPP mostly acted in the gas phase and left very little residual char from combustion. Therefore, the PHRR is reduced, but not much, and it was assumed that the significant decreases in TSR and THR may be due to the prominent gas-phase mechanism of TPP and the partly condensed phase action of TPP and/or PUF (Figure 11). The data also allowed further assumptions that there was a crosslinker between the pyrolytic products of PU-DZS and TPP to form residual char. However, these char layers are not stable enough to undergo further thermo-oxidative degradation, and therefore, very little residual char can be obtained after the cone calorimeter test.

## 4. Conclusions

Rigid PUF was prepared successfully by the reaction of MDI with a novel oligo-ester-ether-diol obtained from the glycolysis of waste PET by DEG by using ZnSO_4_·7H_2_O under microwave irradiation. The liquid state of the oligodiol was only obtained when the minimum DEG/PET molar ratio of 2.5/1.0 was used. The oligodiol structures were confirmed by LC-MS and NMR analysis. For the glycolysis of PET, the results prove not only the transesterification of DEG with the ester groups of PET but also the concurrent ether formation between alcohol end groups. 

Three PUF samples, PU-DZA, PU-DZS, and PU-DZS-TPP_25_, were prepared and showed clear T_g_s and T_m_s of the soft oligo-ester-ether-diol segments in the chain. The PU-DZS-TPP_25_ sample meets the demands of density and compression strength for commercial PUF and has an excellent flame retardancy and high thermal stability. The PUF passed a UL-94 V-0 rating with a 10 wt % TPP loading, and the LOI value reached 21%; the PHRR, THR, and TSR were reduced and in good agreement with the UL94 and LOI results. All starting materials including the PET waste, DEG, MDI were completely transformed into the final PUF, except for the small volatile material present during PET glycolysis. In this study, the PET waste contributed up to 20.5% of the final PUF product; in other words, a high percentage of PET was recycled. Consequently, the reported procedure for PET chemical recycling is more effective and environmentally friendly.

## Figures and Tables

**Table 1:** UL-94 and LOI results for PUF-DZS and PUF-DZS containing TPP.

Samples	UL-94		LOI (%)
	UL-94 HB	UL-94 V	
PU-DZS	Not achieved v = 120 mm/min	No rating	17
PU-DZS-TPP_5_	Not achieved v = 75 mm/min	No rating	
PU-DZS-TPP_15_	HB	V-1	
PU-DZS-TPP_25_	HB	V-0	21
